# Processes Driving the Adaptive Radiation of a Tropical Tree ( *Diospyros* , Ebenaceae) in New Caledonia, a Biodiversity Hotspot

**DOI:** 10.1093/sysbio/syv076

**Published:** 2015-10-01

**Authors:** Ovidiu Paun, Barbara Turner, Emiliano Trucchi, Jérôme Munzinger, Mark W. Chase, Rosabelle Samuel

**Affiliations:** ^1^ Department of Botany and Biodiversity Research, University of Vienna, 1030 Vienna, Austria;; ^2^ IRD, UMR AMAP, 34398 Montpellier, France;; ^3^ Jodrell Laboratory, Royal Botanic Gardens, Kew, TW9 3AB Surrey, UK; and; ^4^ School of Plant Biology, University of Western Australia, Crawley, WA 6009 Australia

**Keywords:** Adaptive radiation, *Diospyros*, hybridization, New Caledonia, RAD-sequencing, soil adaptation

## Abstract

Due to its special geological history, the New Caledonian Archipelago is a mosaic of soil types, and in combination with climatic conditions this results in a heterogeneous environment across relatively small distances. A group of over 20 endemic species of *Diospyros* (Ebenaceae) has rapidly and recently radiated on the archipelago after a single long-distance dispersal event. Most of the *Diospyros* species in the radiating group are morphologically and ecologically well differentiated, but they exhibit low levels of DNA variability. To investigate the processes that shaped the diversification of this group we employed restriction site associated DNA sequencing (RADseq). Over 8400 filtered SNPs generally confirm species delimitations and produce a well-supported phylogenetic tree. Our analyses document local introgression, but only a limited potential for gene flow over longer distances. The phylogenetic relationships point to an early regional clustering among populations and species, indicating that allopatric speciation with respect to macrohabitat (i.e., climatic conditions) may have had a role in the initial differentiation within the group. A later, more rapid radiation involved divergence with respect to microhabitat (i.e., soil preference). Several sister species in the group show a parallel divergence in edaphic preference. Searches for genomic regions that are systematically differentiated in this replicated phenotypic divergence pointed to loci potentially involved in ion binding and cellular transport. These loci appear meaningful in the context of adaptations to soil types that differ in heavy-metal and mineral content. Identical nucleotide changes affected only two of these loci, indicating that introgression may have played a limited role in their evolution. Our results suggest that both allopatric diversification and (parapatric) ecological divergence shaped successive rounds of speciation in the *Diospyros* radiation on New Caledonia.

Biogeographic regions with significant species richness (i.e., biodiversity hotspots; Willis et al. 2007 ), and ecological-opportunistic, explosive diversifications of one lineage into an array of new species (i.e., adaptive radiations; Gavrilets and Losos 2009 ) promise to provide tremendous evolutionary insights. The accumulation of biodiversity within a particular group or a geographic area opens the way to integrative studies into the drivers of evolutionary opportunity and the ecological and genetic limits of adaptation ( Losos 2010 ). However, the complexity of evolutionary relationships in such cases poses significant challenges to phylogenetic inference ( Glor 2010 ), which is required as starting point for further studies of the evolutionary processes shaping observed species richness.

Adaptive radiations are generally linked to ecological opportunity, often starting from long-distance dispersal events, when an alien lineage takes advantage of an array of newly formed or previously unfilled niches ( Glor 2010 ). Mathematical models of adaptive radiation point to a rapid burst of speciation events soon after the beginning of radiations ( Gavrilets and Vose 2005 ), in time resulting in overshooting (decreasing speciation and/or increasing extinction rates; Gavrilets and Losos 2009 ). However, in the case of isolated areas (e.g., oceanic islands) the initial founder population is likely to have an extremely small effective size, with only limited variation that can be selected and potentially result in novel and divergent adaptations. Due to low levels of genetic variation and small population size, the evolution of island biotas is hence intuitively expected to be shaped by neutral processes rather than natural selection ( Ohta 2002 ). In particular for organisms with long generation time, such as trees, the initial stages of radiation are expected to be notably difficult, and the evolutionary processes shaping such radiations are little understood.

We analyze here a putative radiation of a tropical tree ( *Diospyros* , Ebenaceae) on New Caledonia, an archipelago in the Pacific, about 1600 km east of Australia. The continental part of New Caledonia separated from East Gondwana in the late Cretaceous (ca. 80 Ma; McLoughlin 2001 ). During the Paleocene and early to late Eocene (65–37 Ma), the Archipelago was submerged, and a thick layer of oceanic sediments accumulated over its surface ( Pelletier 2006 ). After it re-emerged in the late Eocene, a heavy-metal rich oceanic material covered the land area, and due to later erosion around one-third of the main island is still overlaid today with ultramafic substrates (i.e., heavy metal rich). Due to this complex geological history, New Caledonia displays a mosaic of soil types ( Pelletier 2006 ; Maurizot and Vendé-Leclerc 2009 ), and in combination with its diverse terrain and climate this results in a heterogeneous environment across a fairly small area (i.e., roughly 60 × 400 km for the main island; Fig. 1 ). As a result, the Archipelago hosts an unusually concentrated level of biodiversity, and it is often cited as one of the areas with the highest rate of plant endemism ( Myers et al. 2000 ; Mittermeier et al. 2004 ; Kier et al. 2009 ), with some genera and even families unique to these islands ( Morat et al. 2012 ). Due to mining and agriculture, this biodiversity is under continuous human threat and New Caledonia is one of the regions with the highest predicted land loss as a result of climate change-driven increase of sea levels ( Bellard et al. 2013 ).

**Figure 1.**
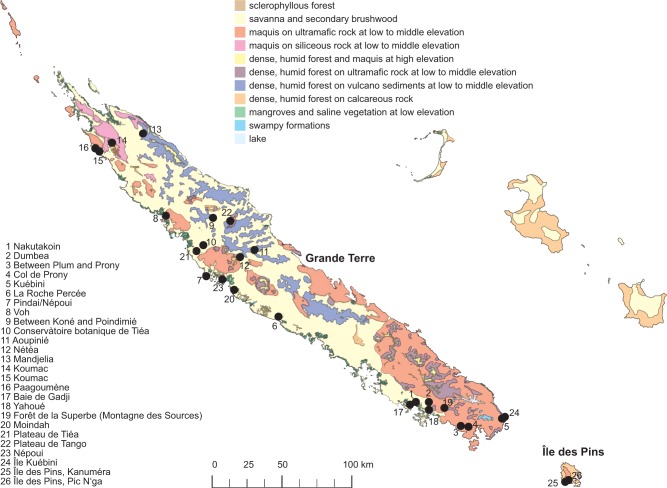
Map of New Caledonia indicating the 26 sampling localities for this study. Numbered dots indicate sampling sites (see also Table 1 ). The colors indicate the vegetation types ( Jaffré et al. 2012 ) according to the legend insert.

*Diospyros* is a large pantropical genus of woody plants that colonized New Caledonia via long-distance dispersal at least four times during the last 25 myr ( Duangjai et al. 2009 ). Three of these colonization events gave rise to only a small set of species (one to five species each; Turner et al. 2013a ). However, another event gave rise to a clade of 24 closely related species that take advantage of all vegetation types on the Archipelago (except mangroves; Turner et al. 2013b ). Most of these closely related species are morphologically and ecologically clearly differentiated, and only a few of them occur in local sympatry. Several of the species are point endemics. Due to extremely low levels of sequence divergence at several plastid and low-copy nuclear regions, the relationships between these species have previously proven difficult to clarify, even with extensive data sampling ( Duangjai et al. 2009 ; Turner et al. 2013a , 2013b ). However, delimitation of most species has been confirmed with amplified fragment length polymorphisms (AFLPs; Turner et al. 2013b ). Previous phylogenetic studies ( Duangjai et al. 2009 ; Turner et al. 2013a ) have shown this group to be related to species found on islands of the Indian Ocean and Hawai'i. Molecular dating based on combined plastid and low-copy nuclear DNA sequence data showed that the lineage forming this group of *Diospyros* species split from its sister species around 9 Ma, with the majority of species diverging within the last 2 myr ( Turner et al. 2013a ). Taking into consideration that these are woody plants with generation time of ca. 7 years ( Verdú 2002 ), we can estimate there have been fewer than 1.3 million generations since the first members of the group arrived on the Archipelago. Such a time frame may be insufficient for genome-wide genetic differentiation among these species to have accumulated, especially at the scale of a relatively small area where interspecific gene flow may have been frequent.

Phenotypic novelties, reproductive isolation, and adaptation to environmental conditions do not necessarily depend on large-scale genetic alterations; they can be due to divergence at only a few loci ( Wu 2001 ; Lexer and Widmer 2008 ; Kane et al. 2009 ). Finding such relatively small differences within non-model genomes is challenging. Recently developed high-throughput DNA sequencing technologies and analysis algorithms provide an opportunity to study in detail genome-wide variability across groups of individuals, adding significant power to evolutionary investigations. In particular, restriction site associated DNA sequencing (RADseq; Baird et al. 2008 ) has been suggested as a powerful tool for interspecific comparisons across a large number of loci in non-model organisms ( Cariou et al. 2013 ), delivering clear phylogenetic information even in the presence of incomplete lineage sorting ( Wagner et al. 2013 ). We use RADseq here to attempt to resolve phylogenetic relationships among 21 (out of 24) diploid *Diospyros* species that recently and rapidly radiated on New Caledonia in order to identify the evolutionary processes that shaped this radiation.

## M aterials and M ethods

### Taxon Sampling and DNA Isolation

Leaf material from New Caledonian *Diospyros* species was collected on Grande Terre and Île des Pins at 27 localities ( Fig. 1 , Table 1 ) and stored in silica gel. Herbarium vouchers are deposited in the herbaria of Nouméa (NOU), the University of Montpellier (MPU), and the University of Vienna (WU; see Table 1 ). Whenever possible, we aimed to investigate at least two individuals per sampling locality and a minimum of three individuals per species. Our data set contains 84 individuals from 39 populations ( Fig. 1 , Table 1 ), including representatives of 21 species of New Caledonian *Diospyros* that have been previously shown to have radiated rapidly after a single long-distance dispersal event ( Turner et al. 2013a ). One of the studied species (collected at Île des Pins, Pic N'ga) could not be unambiguously identified—due to the absence of diagnostic reproductive organs at the time of collection—and is referred to as *D* .sp. Pic N'ga.

**Table 1.** Details of the samples, populations, and species included in the present study

Taxon	Sample ID	Sampling location ^a^	Voucher ^b^
*D. calciphila* F.White	BT313 BT314 BT317	25, littoral forest	JM6650, JM6653 (MPU, NOU, P)
*D. cherrieri* F.White	BT276 BT278	20, dry forest	NOU054492 NOU054008
*D. cherrieri*	BT293 BT294	23, dry forest	NOU079547
*D. erudita* F.White	BT280 BT281	21, dry forest	WU062858, Chambrey & Turner 22 (NOU)
*D. flavocarpa* (Vieill. ex P.Parm.) F.White	BT129 BT130	9, humid mountain forest	JM6625 (NOU)
*D. flavocarpa*	BT156 BT157	11, dense humid mountain forest	JM6632 (NOU)
*D. glans* F.White	BT093 BT094	5, forest near river	NOU022860
*D. impolita* F.White	BT102 BT103 BT105	6, mesophyll forest near beach	NOU019538
*D. inexplorata* F.White	BT308 BT310 BT311	24, littoral forest	NOU005818
*D. labillardierei* F.White	BT122 BT125	9, river edge in mountain forest	JM6624 (NOU)
*D. labillardierei*	BT178 BT182	12, river edge	(NOU031346)
*D. minimifolia* F.White	BT131 BT135	10, dry forest	NOU019556
*D. minimifolia*	BT232 BT233	17, mesophyll forest near beach	NOU019554
*D. minimifolia*	BT263 BT269	20, dry forest	NOU079549, WU062872 NOU054493
*D. pancheri* Kosterm.	BT028 BT031 BT035	3, forest near road	JM6619, JM6620 (NOU)
*D. parviflora* (Schltr.) Bakh.	BT038 BT041 BT042	4, wet forest	
*D. parviflora*	BT147 BT148	10, forest near river	JM6630 (NOU)
*D. parviflora*	BT187	13, mountain forest	JM6636 (NOU)
*D. parviflora*	BT250	19, humid forest at low elevation	tree no. 23109
*D. parviflora*	BT289 BT290 BT291	22, mountain forest	NOU079550
*D. perplexa* F.White	BT004	1, mesophyll forest	JM6611, JM6613 (NOU)
*D. pustulata* F.White	BT111 BT112	7, dry forest	
*D. pustulata*	BT137 BT140	10, dry forest	JM6629 (NOU)
*D. pustulata*	BT259 BT261 BT265 BT268	20, dry forest	WU062855 NOU079544, WU062870, NOU079548, WU062871 NOU053999
*D. revolutissima* F.White	BT117 BT120	8, maquis	NOU023189
*D. revolutissima*	BT219 BT221	16, maquis	JM6640 (NOU)
*D. tridentata* F.White	BT203 BT206 BT207	14, dry forest at low elevation	JM6639 (NOU)
*D. trisulca* F.White	BT185 BT192 BT199 BT201	13, mountain forest	NOU031344JM6637 (NOU)
*D. umbrosa* F.White	BT176 BT177	12, dense humid forest	JM6635 (NOU)
*D. umbrosa*	BT197	13, mountain forest	
*D. umbrosa*	BT246 BT247	19, humid forest at low elevation	NOU023234
*D. veillonii* F.White	BT224 BT226 BT227	17, mesophyll forest near beach	NOU019582
*D. vieillardii* (Hiern) Kosterm.	BT025 BT026	2, forest near river	JM6618 (NOU)
*D. vieillardii*	BT088 BT100	5, forest near river	
*D. vieillardii*	BT215 BT217	15, maquis	NOU023242
*D. vieillardii*	BT286	21, dry forest	
*D. yahouensis* (Schltr.) Kosterm.	BT238 BT239	18, mesophyll forest	P00057340
*D* . sp. Pic N'ga	BT318 BT320 BT323	26, maquis	JM6065 (NOU)

^a^ The identification number of sampling localities are following Figure 1 .

^b^ Voucher-Codes: JMXXXX: collection number J. Munzinger; Tree No. XXXXX: Tree of New Caledonian Plant Inventory and Permanent Plot Network (NC-PIPPN, Ibanez et al. 2014 ); NOUXXXXXXX: Herbarium accession number of Noumea herbarium (NOU); WUXXXXXX: Herbarium accession number of the Herbarium of the University Vienna (WU); P: Herbarium of the Natural History Museum Paris; MPU: Herbarium of the University of Montpellier.

DNA extractions performed with a modified sorbitol/high-salt CTAB (cetyltrimethylammonium bromide) method ( Tel-Zur et al. 1999 ) were already available from a previous study ( Turner et al. 2013b ). As we observed significant differences between standard Nanodrop (Thermo Scientific) and Quant-It Pico-Green (Life Technologies) quantifications of the DNA samples, these have been purified using the NucleoSpin gDNA clean-up kit (Macherey-Nagel), according to the manufacturer's protocol.

### RADseq Library Preparation

By using an average genome size in the target group of 1C = 1.9 pg ( Turner et al. 2013a ) and the RAD counter, available from www.wiki.ed.ac.uk/display/RADSequencing/Home ; last accessed October 15, 2015, we have estimated that 60 individually barcoded samples can be pooled together when using the *SbfI* restriction enzyme (New England Biolabs). Due to uneven representation of individuals within the library, a second RAD library was later prepared in order to increase the number of reads of selected samples to a minimum 1 million high-quality read pairs per individual and to add 24 additional individuals. The RAD libraries were prepared using a protocol adapted from Baird et al. (2008) with modifications. We started with 300 ng DNA per individual and used double barcoding to decrease the number of different adapters necessary. The six-base-pair P5 barcodes and, respectively, the four-base-pair P7 barcodes were chosen to differ by at least three bases from each other to avoid erroneous assignment due to sequencing error. We ligated 200 mM P5 adapters to the restricted samples overnight at 16°C. Groups of samples barcoded with different P5 barcodes were pooled, and sheared by sonication with a Bioruptor Pico (Diagenode) to achieve an average size of ca. 400 bp, using two cycles of 55 s “on” and 55 s “off” at 6°C. In order to obtain the optimal fragment sizes for Illumina sequencing, we have performed a left and right size selection with SPRIselect (Beckman Coulter), by using ×0.7 and, respectively, ×0.55 volume of SPRI reagent to sample. After ligating P7 adaptors, all samples were pooled after quantification, so that each sample would be equally represented. To remove unwanted primer dimers two size selections on the left side with ×0.65 volume of SPRI reagent to sample have been finally performed: once before the 18 cycles PCR amplification in the Phusion Master Mix (Thermo Fischer Scientific), and again afterwards. The libraries were sequenced on an Illumina HiSeq at CSF Vienna ( http://csf.ac.at/facilities/next-generation-sequencing/ ; last accessed October 15, 2015) as 100 bp paired-end reads.

### Filtering SNPs from RADseq Data

The libraries were demultiplexed into individual samples according to the respective barcode combinations by allowing for single errors at the barcodes using the RADpools module of the RADtools v. 1.2.4 package ( Baxter et al. 2011 ). The 84 individual files have been further imported in the CLC Genomic Workbench v. 6.5 (Qiagen) and trimmed/filtered to retain only full-length (i.e., 94 bp after barcode trimming) reads, free of any adaptor sequence and with all bases of a quality Phred score ⩾30 . The final high-quality filtered and demultiplexed data set contained over 160 million pairs of reads.

The forward reads were further used for running denovo_map.pl script of STACKS v. 1.12 ( Catchen et al. 2011 ). To find the best settings for STACKS, we first varied the value of the minimum number of identical reads required for a stack to be formed (i.e., the setting “ m ”) from 5 to 15, by allowing one base pair difference between loci when processing one individual (i.e., the setting “ M=1 ”) and when building the catalog (i.e., setting “ n=1 ”). We have chosen the value of m=13 as the best for our data because it delivered the most polymorphic stacks with less than 10 single nucleotide polymorphism (SNP) positions (in order to avoid any pooled paralogs in the same locus) that are covered by data in at least 90% of individuals (i.e., with data present for at least 75 individuals, to avoid artificially splitting individual loci). Further, for the value of m=13 we have run additional tests by varying the value of “ M ” from 1 to 4 and the value of “ n ” from 0 to 6 (Supplementary Table S1, available on Dryad at http://dx.doi.org/10.5061/dryad.62247 ). The final combination of settings was chosen based on the criteria above for m=13 , M=1 , and n=1 .

The deleveraging algorithm of stacks has been left on in order to split loci merged incorrectly and remove highly repetitive sequences from further analyses. To avoid retention of any merged paralogs, the loci showing 10 or more SNPs have been blacklisted in further analyses by filtering them out using the export_sql.pl script from STACKS. The SNP data have been extracted by using the populations script of STACKS. By retaining SNPs from loci covered in at least 75 individuals with a maximum of 9 polymorphic nucleotide positions, we obtained a data matrix containing 8488 concatenated SNPs (hereafter data set D1), which has been further used for phylogenomic analyses. In an additional filtering step we retained only one SNP per locus (data set D2 hereafter, including 1506 SNPs) to minimize linkage in the data, and we finally filtered out all apomorphic SNPs (i.e., distinguishing only single individuals from the rest) to obtain a reduced matrix of 791 SNPs (hereafter data set D3). Data matrices including loci with more missing data (up to 20% and, respectively, up to 50%) per locus resulted in less resolved phylogenetic trees and have been discarded (not shown).

### Phylogenomic Analyses

To study the phylogenetic relationships between the sampled *Diospyros* individuals, we used maximum parsimony (MP), maximum likelihood (ML) and Bayesian inference (BI) on data set D1. For BI and molecular dating, the program BEAST v1.7.5 ( Drummond et al. 2012 ) was run on the CIPRES Science Gateway ( http://www.phylo.org/portal2/ ; last accessed October 15, 2015; Miller et al. 2010 ). Estimation of evolutionary models was conducted with jModelTest v2.1.4 ( Darriba et al. 2012 ) that indicated the transversional model (TVMef; Posada 2003 ) with equal frequencies modeled with a gamma distribution and a proportion of invariable sites (TVMef +Γ+ I) to be the best fit to our data. We used a relaxed uncorrelated log-normal clock model to keep the age estimations flexible ( Drummond et al. 2006 ). Because of the relatively young age of the investigated *Diospyros* group, and therefore a low proportion of lineage extinction expected, we opted for a simple Yule speciation model ( Yule 1925 ; Gernhard 2008 ). Priors for substitution rates between bases (gamma shape 10), alpha (gamma shape 10), and p-inv (uniform) were inferred by jModelTest v2.1.4 ( Darriba et al. 2012 ). Two independent Metropolis-coupled Markov chain Monte Carlo (MCMC) analyses each with 20 million generations were run sampling every 1000th generation. The initial 10% of trees obtained from each MCMC run were removed as burn in; the remaining trees of both runs were used to calculate a maximum clade credibility tree. With BEAST we also conducted molecular dating. Because no fossils pertaining to this New Caledonian *Diospyros* group are available, dating estimates were obtained by secondary calibration: we took into account the age of the split between *Diospyrosvieillardii* and the rest of the group (7.2 Ma) so that it conforms to a previous date obtained for this split ( Turner et al. 2013a ). We constructed the priors (log normal shape) by defining this age as a minimum (i.e., no fixed upper limit), to accommodate the uncertainty of this estimate.

Parsimony analyses were run with PAUP* v4b10 ( Swofford 2003 ) on data set D1 using a heuristic search with stepwise addition, random sequence addition (1000 replicates) and tree-bisection-reconnection. To estimate clade support, bootstrapping with 1000 replicates was performed. We rooted the tree obtained with *D. vieillardii* , according to earlier results ( Turner et al. 2013a ).

Trees were also estimated using data set D1 within a ML framework using RAxML 8.1.3 ( Stamatakis 2014 ). We used the BFGS (Broyden-Fletcher-Goldfarb-Shanno) method to optimize GTR rate parameters, the gamma model of rate heterogeneity, and 1000 rapid bootstrap inferences with a subsequent thorough ML search. The results were visualized with FigTree 1.4 (available from http://tree.bio.ed.ac.uk/software/figtree/ ; last accessed October 15, 2015).

Finally, we inferred a species tree on data set D2 using the Bayesian coalescent method implemented in SNAPP ( Bryant et al. 2012 ). As SNAPP is designed to deal with biallelic unlinked data, we extracted one SNP at random per locus. Given the average density of our RAD loci (less than 1/Mb), the probability of any of those SNPs to be in linkage is low. Nevertheless, a small amount of linkage among markers is not expected to bias the analysis ( Bryant et al. 2012 ). For the SNAPP analyses, the individuals were a priori grouped to species, apart from those species that were shown to form multiple clusters in the MP/ML/BI trees, where the grouping used followed sampling localities. Two localities where only single individuals were available were excluded from the analyses (i.e., *Diospyrosperplexa* from L1 and *Diospyrosparviflora* from L13). We set a prior for $\theta =4\mu N_{\text{e}}$ as a gamma distribution with α=1.15 and β=10,000 ( $\text{mean}=1.15\times 10^{\text{-4}})$ . We ran two independent MCMC chains for $5\times 10^{\text{6}}$ generations, each sampling every 100th generation. Burn-in was visually determined and convergence among different chains checked in Tracer 1.6. We also checked that all ESSs were more than 100 across combined runs, with most of them more than 200. Trees resulting from the analysis were then visualized in DensiTree ( Bouckaert 2010 ). We summarized all trees in a set of *consensus* trees, where each consensus tree is calculated from the subset of trees sharing the same topology averaging the branch length over this subset. A large number of consensus trees denotes a high degree of uncertainty in topology.

### Genetic Clustering and Patterns of Reticulation

Within closely related groups, representation of relationships as networks rather than bifurcating trees appears to be more appropriate ( Huson and Scornavacca 2011 ) to account for the potential presence of hybridization. We used SplitsTree v.4.12.6 ( Huson and Bryant 2006 ) to create a network based on Hamming distances ( Hamming 1950 ) and data set D1. The simple calculation method of Hamming distance was considered appropriate for the RADseq-derived SNPs data set that lacks indels after processing with STACKS.

To investigate the higher-level clustering of the included individuals and potential admixture between different groups we used Structure v2.3.3 ( Pritchard et al. 2000 ; Hubisz et al. 2009 ) on data set D3. We ran the program separately for (i) all individuals analyzed and (ii) the individuals forming the larger cluster in the first analysis (i.e., the RR clade as defined in Results). Both analyses have been performed at the Lifeportal of the University of Oslo for K=1 –10, each with 10 replicates, and a model based on admixture and independent allelic frequencies. Each run had 1 million iterations with 10% additional burn in. The calculation of ΔK ( Evanno et al. 2005 ; Supplementary Fig. S3, available on Dryad at http://dx.doi.org/10.5061/dryad.62247 ) was performed with Harvester ( Earl and vonHoldt 2012 ). To avoid any stochastic aspect of the process, we have produced a permuted matrix from the 10 replicates of the best K value with Clumpp v1.1.2 ( Jakobsson and Rosenberg 2007 ) in the greedy algorithm and 1000 repeats. The graphical display of Structure results was prepared with Distruct v1.1 ( Rosenberg 2004 ).

Historical relationships inferred with a population graph analysis that allows both population splits and migration events were constructed with TreeMix 1.12 ( Pickrell and Pritchard 2012 ) on data set D2, but including only populations from the RR group, plus *Diospyroscherrieri* , used as outgroup (Supplementary Fig. S4, available on Dryad at http://dx.doi.org/10.5061/dryad.62247 ). The individuals were grouped according to sampling localities and species (excluding localities represented by single individuals, except for *D. parviflora* L19 which was pooled together with individuals from the same species from L4 according to above presented results). We have added migration edges stepwise with up to 10 events and inspected the results for consistency between the different runs.

### Patterns of Niche Divergence

To investigate how niche specialization contributed to diversification, we attempted to reconstruct ancestral preference for habitats along the inferred Bayesian phylogeny. We employed for this purpose ML inferences of niche evolution with the Lagrange dispersal, extinction, and cladogenesis ( Ree and Smith 2008 ) model implemented in RASP v.3.02 ( Yu et al. 2015 ). We carried out two analyses: one for the evolution of climatic niches (i.e., humid vs. dry) and one for edaphic niches (i.e., ultramafic, volcanic, limestone, schist, and, respectively, serpentine). We did not apply any constraints on the connectivity between the different niches. As all extant taxa are confined to a single soil type, and for simplicity, we allowed for ancestral ranges combining a maximum of two edaphic types.

The phylogenetic trees obtained pointed to several sister clades comprising individuals with divergent preference for ultramafic versus volcanic soils: *Diospyrosflavocarpa* / *Diospyrosumbrosa* ; *D. parviflora* (L10)/ *D. parviflora* (L22); *Diospyrosyahouensis* + *D. perplexa* / *Diospyrospancheri* ; *Diospyrosminimifolia* (L17)/ *D* . sp. Pic N'ga. We searched for RAD regions that contained SNPs with pairwise $F_{\text{ST}}$ values over 0.5 at least for two pairs of sister species with divergent soil preferences. We then made use of the paired-end RADseq data and assembled mini-contigs of the candidate stacks by extracting a list of reads for each locus with Sort_read_pairs.pl from STACKS, sorting reads from FastQ files with Fastq.filter.pl (L. M. Rodriguez, unpublished data, available from http://enveomics.blogspot.co.at/2013/04/fastqfilterpl.html ; last accessed October 15, 2015) and assembling each set in the CLC Genomic Workbench (QIAGEN), with automatic optimization of the word and bubble sizes and updating the contigs after mapping back the reads. We finally performed functional annotation analyses for the obtained contigs using Blast2GO v. 3.1.3 (BioBam; Götz et al. 2008 ) with default settings (i.e., blastx searches against nr) and integrating GO ( www.geneontology.org ; last accessed October 15, 2015), KEGG ( www.genome.jp/kegg ; last accessed October 15, 2015), and InterProScan ( http://www.ebi.ac.uk/Tools/webservices/services/pfa/iprscan5_soap ; last accessed October 15, 2015) information in our results. The biological meaning of the set of sequences has been investigated with the combined graph option of Blast2Go by visualizing only categories with a node score ⩾2.00 . Further, a Fisher's exact test has been run with Blast2Go to investigate the significance of any GO term enrichment in the potential adaptive loci against a reference group of 1000 RADseq loci assembled in a similar way.

## R esults

### Phylogenomic Analyses

After demultiplexing, trimming and filtering the raw 100 bp reads from two RADseq libraries, we retained on average 1.9 ± 0.7 million high-quality pairs of reads per individual. After parameter optimization as described above, the *de novo* assembly pipeline of STACKS produced 37,336 loci (excluding any tags present in only one individual).

**Figure 2.**
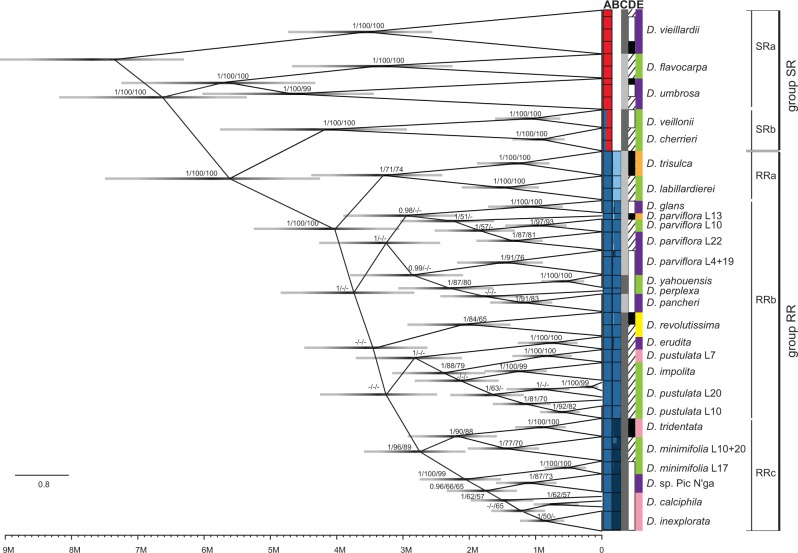
Phylogenetic tree of the radiating *Diospyros* species on New Caledonia derived from Bayesian inference. For simplicity, individuals are collapsed to species or population level wherever possible. Node bars indicate the 95% confidence interval for the age of the corresponding node. A time scale is given at the bottom of the figure. Numbers at nodes detail BI posterior probabilities greater than 0.95/ML confidence over 50%/bootstrap support values over 50%. The vertical bars indicate: a) Structure results for all individuals for K=2 ; b) Structure results for the subset of individuals from group RR, for K=3 ; c) the preference for climate type, generally following a E–W separation—light gray for humid, dark gray for dry; d) the geography, generally following a N–S separation—black for north, hashed area for center, and white bars for south; and e) for substrate-type preference—lemon green for volcanic rock, dark purple for ultramafic rock, orange for schist, pink for limestone, and yellow for serpentine. L. refers to sampling location given in Fig. 1 and Table 1 .

The MP (Supplementary Fig. S1, available on Dryad at http://dx.doi.org/10.5061/dryad.62247 ), ML (Supplementary Fig. S2, available on Dryad at http://dx.doi.org/10.5061/dryad.62247 ), and BI ( Fig. 2 ) analyses resulted in generally consistent topologies, pointing to two major groups of species: one older grade of more slowly radiating species (SR hereafter) that are more divergent, and a younger clade of more rapidly radiating species (RR hereafter). The backbones of the other trees are slightly less supported than that of the BI ( Fig. 2 ).

Apart from *D. minimifolia* , *D. parviflora* , and *Diospyrospustulata* , all other species form highly supported clusters in the BI phylogeny. The individuals of the same population always group together and are well supported, except for one population of *D. pustulata* (location 20). Further, the sampled individuals of *Diospyroscalciphila* form a paraphyletic group, embedding *Diospyrosinexplorata* individuals despite growing on different islands. We do not observe any major grouping related to ecological factors like soil type or climate, but sister species often show divergent ecological preferences ( Fig. 2 ). The molecular clock analysis resulted in a slightly older age for the split of *D. vieillardii* from the rest of the group, estimated at 7.4 Ma, with a wide 95% confidence interval of 2.7 Ma. The next divergence (i.e., *D. flavocarpa* / *D. umbrosa* from the rest of the species) took place around 6.6 Ma. The lineage forming *D. cherrieri* and *Diospyrosveillonii* separated from the rest around 5.6 Ma. The RR group started to diversify around 4 Ma. The RRc clade is a young group, about 2.7 myr old. Most speciation events seem to have taken place in this group between 3.5 and 1.5 Ma.

The MP analysis resulted in 31 equally parsimonious trees (Supplementary Fig. S1, available on Dryad at http://dx.doi.org/10.5061/dryad.62247 ). The phylogenetic relationships between the earlier diverged lineages (group SR) are the same as in the BI genealogy. The relationships between the clades within group RR differ slightly between MP (Supplementary Fig. S1, available on Dryad at http://dx.doi.org/10.5061/dryad.62247 ) and BI ( Fig. 2 ). The general topology of the ML tree (Supplementary Fig. S2, available on Dryad at http://dx.doi.org/10.5061/dryad.62247 ) inferred with RAxML is also similar to that of the BI.

**Figure 3.**
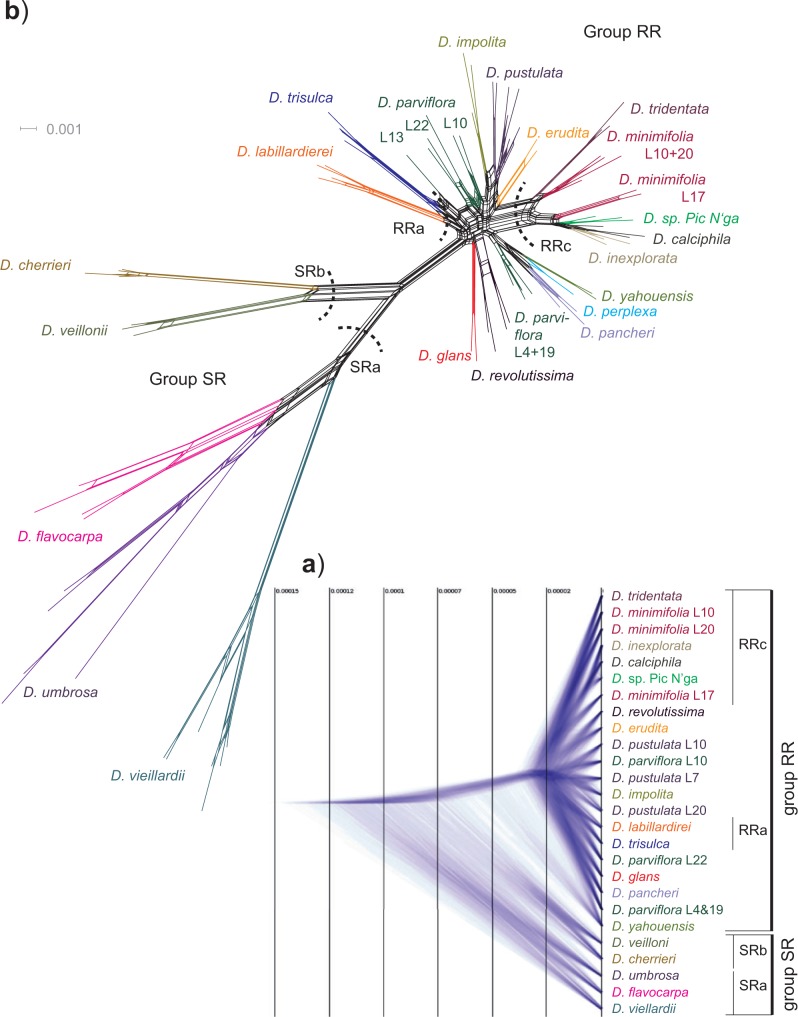
a) Species trees generated by SNAPP analysis. The distances on the horizontal axis are relative measures of substitutions per site. The nodes are positioned as a star tree to highlight the timing of species radiation. Time on the grid is scaled to the number of mutations. b) NeighbourNet based on Hamming distance. L. refers to sampling locations given in Fig. 1 and Table 1 .

The SNAPP results ( Fig. 3 a) reveal a species tree of similar major topology to the BI tree, but with several marked differences. Generally, the SNAPP analyses seems to have less resolving power than the BI, most probably due to more informative characters present in the matrix used for the latter. Whereas the composition of the SR and RR groups was found to be constant between the two analyses, SNAPP unexpectedly identifies *D. yahouensis* as the sister species to the rest of the RR group. It also points to a fairly significant time gap between any earlier split and the recent rapid radiation of the RR group. In fact, the relative age of the radiation of the RR group in comparison to the time of divergence since *D. vieillardii* split from the rest of the species seems even younger in the SNAPP analyses in comparison to BI. Due to short internal branch lengths, the relationships between the species of the RR group remain generally unclear and resemble a rapid star-like radiation. However, the RRa and RRc groups that received good support in the BI, MP, and ML analyses are also present in the SNAPP results.

### Genetic Clustering and Patterns of Reticulation

The SplitsTree network ( Fig. 3 b) mirrors the general pattern evident in the phylogenetic analyses. The branches within group SR and between SR and RR groups are significantly longer than those within the RR group, in a ratio more similar to the SNAPP results rather than for BI. The RR group has a reticulate history, indicative of hybridization and/or incomplete lineage sorting. Conflicting information is also apparent at the level of deeper relationships within the SR group.

When including all individuals, Structure identifies two major clades ( Fig. 2 Column a; Supplementary Fig. S3a, available on Dryad at http://dx.doi.org/10.5061/dryad.62247 ), roughly corresponding to the SR and RR groups. However, it shows the presence of potential admixture between the two groups in *D. veillonii* and *D. cherrieri* (SRb subgroup). When run only on the subset of individuals from the RR group, Structure further separates three subgroups ( Fig. 2 Column b; Supplementary Fig. S3b, available on Dryad at http://dx.doi.org/10.5061/dryad.62247 ) with few admixed individuals between them.

We further used ancestry graphs implemented in TreeMix ( Pickrell and Pritchard 2012 ) to identify patterns of divergence and migration within the RR group only, including *D. cherrieri* as an outgroup. The stepwise addition of migration events in the analysis resulted in an incremental, consistent inclusion of additional events up to the sixth migration. With the seventh migration, the events changed significantly, so we considered as final the result including six migrations (Supplementary Fig. S4, available on Dryad at http://dx.doi.org/10.5061/dryad.62247 ). Those six migration events link populations from different climatic conditions (three of six), different soil types (four of six events), and from different regions (five of six events—connecting populations from the center of the island with those in the north or in the south).

### Patterns of Adaptive Divergence

Lagrange analyses (Supplementary Fig. S5, available on Dryad at http://dx.doi.org/10.5061/dryad.62247 ) suggest the most recent common ancestor of all members of this *Diospyros* group that radiated on New Caledonia was most likely a generalist for the amount of humidity, and it might have occupied both ultramafic and volcanic substrates. The generalist lineage had specialist descendants with regard to climate type relatively early, resulting in large clades of extant taxa sharing the same preference for humidity. In contrast, edaphic specialists evolved in general more recently. Surprisingly, apart from multiple transitions from edaphic generalists to specialists, several shifts from specialists to generalists have also been inferred. In addition, specialists to particular substrates are often not each other's closest relatives, but they are rather related to sister taxa adapted to different substrates.

The phylogenetic trees obtained provide evidence that substrate differences exist for several pairs of species, which suggests that ecologically driven isolation could have made a major contribution in driving speciation across the radiating group. We aimed to test if any particular genomic regions (but not necessarily the same exact SNP position) have systematically been affected as a result of positive selection or genetic hitchhiking in divergences between four replicated pairs of sister species (for details see “Materials and Methods”) with distinct preference for ultramafic versus volcanic substrates. We therefore searched for RAD regions that contained SNPs with high fixation index values ( $F_{\text{ST}}>0.5$ ) at least for two pairs of sister species with such replicated divergent soil preferences. We have identified 50 RAD regions (GenBank accession numbers KT587203–KT587252) showing parallel patterns, including four DNA regions that have been found to be significantly different in three of the four pairs of species (see “Materials and Methods”). The majority of the identified SNPs (74.6%) are not fixed differences between species, potentially indicative of polygenic adaptation, as expected for ecological traits (Savolainen et al. 2013). Using the paired-end read information, we have assembled *de novo* larger DNA regions of between 390 and 909 bp for the 50 candidate loci and performed functional annotations for them. Only 26 of the loci received at least 1 blast hit, and 15 of them could be successfully annotated ( Table 2 ). The combined graph analysis of Blast2Go and a Fisher's exact test against a reference group of RADseq loci indicates an enrichment for regions related to transport (i.e., amide transport, peptide transport, and nitrogen compound transport, all at P<0.05 ) and ion binding (at P<0.05 ) ( Table 2 ; Supplementary Fig. S6, available on Dryad at http://dx.doi.org/10.5061/dryad.62247 ).

**Table 2.** Details of the 26 regions with blast hits, which are hypothesized to be recurrently involved in divergent edaphic adaptation

GenBank	SNP bp	SNP pos. ^a^	Sequence description	GOs
KT587204	7/66/72	Out	Protein aluminium sensitive 3	C: plasma membrane; P: response to aluminium ion
KT587205	77	First	UDP-glycosyltransferase 73c3-like	F: transferase activity, transferring glycosyl groups
KT587206	15/48	Out	Translation machinery-associated protein 22	P: translational initiation; F: translation initiation factor activity
KT587208	49/55	Second/second	Probable calcium-binding protein CML23	F: ion binding
KT587250	51/62	Out	DUF616 family protein	—
KT587212	12/78	Third/third	Copia-like retrotransposable	C: membrane; F: nucleic acid binding; F: zinc ion binding; F: anion binding; P: DNA integration; P: ammonium transport
KT587213	6/51	Third/third	Trihelix transcription factor ASIL2-like	C: vacuole; F: chromatin binding; C: nucleus
KT587214	6/45	Out	SNF2 domain-containing family protein	F: DNA binding; F: ion binding; F: helicase activity
KT587216	34/59	Out	Protein NRT1 PTR family–like	P: transport
KT587248	33/63	Out	Myosin-2-like isoform x1	C: cytoskeleton; F: ion binding; C: protein complex; F: cytoskeletal protein binding
KT587249	52/79	—	Uncharacterized locus	F: ion binding; P: tRNA metabolic process; P: cellular amino acid metabolic process; F: ligase activity; P: translation
KT587217	39/47	Out	Ribonuclease 3	C: plastid
KT587218	43/60/90	Out	gag-pol polyprotein	F: nucleic acid binding; P: DNA integration; F: zinc ion binding; C: nucleus
KT587219	12/84	—	e3 ubiquitin-protein ligase pub24-like	F: ubiquitin–protein transferase activity; P: protein ubiquitination
KT587220	42/58	Out	R-recognition motif	—
KT587223	61/92	Out	CBS domain-containing protein mitochondrial	F: adenyl nucleotide binding; P: metabolic process; F: catalytic activity
KT587224	35/80	First/first	EH domain-containing protein 1	C: cell; F: ion binding
KT587225	38/64	Out	Type II inositol-trisphosphate 5-phosphatase FRA3 isoform x1	P: lipid metabolic process; F: phosphatase activity; F: ion binding
KT587226	28/31	Second/second	Uncharacterized protein TCM_034686	—
KT587227	48/72	Out	Hypothetical protein VITISV_008807	P: oxidation–reduction process; F: heme oxygenase (decyclizing) activity; P: heme oxidation; F: transferase activity; P: metabolic process; F: sulfotransferase activity
KT587244	8/43	Third/second	Hypothetical protein PRUPE_ppa021982mg	—
KT587247	24/40	Second/third	SAUR-like auxin-responsive protein	—
KT587234	20/52/86	Out/third/first	Uncharacterized loc101212813	C: nucleus
KT587231	68	—	ABC transporter b family member 19-like	C: integral component of membrane; F: ATP binding; F: xenobiotic-transporting ATPase activity; P: transmembrane transport; F: transmembrane transporter activity; F: ion binding; P: peptide transport; P: xenobiotic transport
KT587237	68/91	Out	Mitogen-activated protein kinase 19	P: signal transduction; F: ion binding; P: cellular protein modification process; P: cellular amino acid metabolic process; F: signal transducer activity; F: kinase activity
KT587245	69/75	Second/second	(+)-Neomenthol dehydrogenase-like isoform x1	F: oxidoreductase activity; P: oxidation–reduction process

^a^ SNP position refers to the position with respect of the reading frame, inferred from the blast result: out—out of ORF, first, second, and third refer to codon positions.

## D iscussion

### Inferring Shallow Phylogenetic Relationships

To resolve the phylogenetic relationships within a rapidly radiating *Diospyros* group on New Caledonia, we employed thousands of SNPs derived from RAD loci assembled *de novo* from Illumina reads. This allowed us to infer a much more completely resolved tree than previous studies using multiple DNA loci ( Duangjai et al. 2009 ; Turner et al. 2013a ) and genome-wide fingerprinting analyses (AFLP; Turner et al. 2013b ). An increase in phylogenetic resolution when using RADseq in comparison with more traditional methods has already been shown for various organisms, for example, in the case of the adaptive radiation of cichlid fishes in Lake Victoria ( Keller et al. 2013 ; Wagner et al. 2013 ) and the radiation of surfperches in the northern Pacific ( Longo and Bernardi 2015 ). Despite the relatively large number of SNPs we obtained, relationships inferred here for *Diospyros* are not always well supported. The reason for this may lie in the limited number of generations since the extreme bottleneck associated with the initial long-distance dispersal event to New Caledonia. This renders a significant portion of the SNPs to have minor allelic variants present in only one to a handful of individuals (i.e., fairly recently evolved variants). This pattern is clearly visible in the phylogenetic trees we obtained, with well-supported terminal clades corresponding to species or just populations.

Additional processes may have blurred the phylogenetic signal in this rapidly radiating group, in particular introgression, which could have been common during some episodes of speciation in this group. Its effects are visible in the SplitsTree network ( Fig. 3 b) as reticulations, the Structure results (Supplementary Fig. S3, available on Dryad at http://dx.doi.org/10.5061/dryad.62247 ) as the presence of admixed individuals, and the migration events inferred with TreeMix (Supplementary Fig. S4, available on Dryad at http://dx.doi.org/10.5061/dryad.62247 ). Incomplete sorting of ancestral polymorphism requires a rich ancestral genetic pool ( van Oppen et al. 2001 ; Maddison and Knowles 2006 ; Glor 2010 ; Lerner et al. 2011 ). Therefore, we regard the latter process as less likely to have significantly affected, on a genome-wide scale, the phylogenetic patterns within this group, which radiated fairly recently after passing through the extreme bottleneck associated with arrival on New Caledonia ( Duangjai et al. 2009 ). The results obtained are also much too structured by taxon and geography to represent this sort of distortion of phylogenetic relationships.

### Early Divergence with Respect to Macrohabitat and Geographic Distance

Grande Terre, the main island of New Caledonia, is split by a mountain range into humid southeastern (2000–4000 mm precipitation per year) and dry northwestern parts (1000 mm precipitation per year) with prevailing winds and rain coming from the southeast ( Maitrepierre 2012 ). The inferred phylogenetic relationships and the analyses of niche evolution point to an initial, but fairly slow, divergence with respect to these climatic conditions along major clades (i.e., the deep splits on the backbone of the phylogenetic tree; Fig. 2 Column c and Supplementary Fig. S5A, available on Dryad at http://dx.doi.org/10.5061/dryad.62247 ). Modeling studies have suggested that divergence with respect to the macrohabitat is indeed the first expected stage for rapid radiations ( Gavrilets and Losos 2009 ; Glor 2010 ), although up to now the empirical evidence for this expectation was limited. In *Diospyros* , this appears to be the result of climatic specialization from a generalist lineage that, however, persists over several cladogenesis events (Supplementary Fig. S5A, available on Dryad at http://dx.doi.org/10.5061/dryad.62247 ). This may argue against a significant contribution of climatic specialization to diversification in this particular group ( Fine et al. 2014 ). In *Diospyros* , this pattern could also be merely the result of allopatric differentiation, with isolation by distance promoted by a geographic barrier between the dry and wet areas that is difficult to cross. A limited dispersal potential of New Caledonian species of *Diospyros* is supported by the fact that most populations cluster separately within each species (see e.g., the MP tree in Supplementary Fig. S1, available on Dryad at http://dx.doi.org/10.5061/dryad.62247 , or the SplitsTree network in Fig. 3 b). Very little is known about pollinators and fruit dispersal in these species; most species are dioecious and have fleshy fruits. The fruits of other *Diospyros* species present on the island are eaten by birds—for example, the fruits of *Diospyrosfasciculosa* (of similar size as the fruits in the radiating group) are dispersed by the red-bellied fruit-dove, *Ptilinopus greyii* ( Tassin et al. 2010 ). We assume the seeds of the radiating group are also dispersed by birds, but the genetic evidence is consistent with limited gene flow between populations.

We further observe that several intermediate-level splits follow a regional separation ( Fig. 2 Column d), with relatively large groups of species/populations either confined to the northern, the central or the southern portion of the island. For example, the related *Diospyroserudita* , *Diospyrosimpolita* , and *D. pustulata* (from the RRb group) are all found in the middle western part of Grande Terre in dry vegetation (maquis or sclerophyllous forests). A similar regional clustering pattern is observed for *D. pancheri* , *D. yahouensis* , *D. perplexa* , and part of *D. parviflora* ; all were collected in southern part of Grande Terre. These examples support the idea that dispersal over long geographic distances is inefficient in this group, which results in a pattern of isolation by distance.

### Late Divergence with Respect to Microhabitat

In New Caledonia, *Diospyros* species are found in all kinds of vegetation ( Fig. 1 ) except mangroves. However, the species that are elements of similar vegetation types are in general not closely related, a phenomenon that is characterized as phylogenetic niche convergence ( Webb et al. 2002 ). Such a pattern can be the result of niche filling ( Jakob et al. 2010 ), with close relatives adapting to different habitats to avoid highly similar competitors, whereas taxa from distinct clades are different enough to explore diverse resources even in sympatry. For example, typical littoral vegetation along the New Caledonian coasts is represented by species such as *D. calciphila* , *D. inexplorata* (both RRc in Fig. 2 ), *D. impolita* (RRb), and *D. veillonii* (SRb). On the other hand, *D. cherrieri* (SRb), *D. minimifolia* , *Diospyrostridentata* (both RRc), *D. perplexa* , and *D. pustulata* (both RRb) are found in sclerophyll forests on the dry western coast of Grande Terre. Especially when considering multispecies localities (e.g., L10, L13, and L20; Table 1 ), sympatric species are only distantly related.

In contrast, most sister species relationships seem to have been shaped by ecologically driven isolation, in particular with respect to edaphic specialization ( Fig. 2 Column e). Indeed, we find a plenitude of independent edaphic shifts (Supplementary Fig. S5b, available on Dryad at http://dx.doi.org/10.5061/dryad.62247 ) that occur regionally ( Fig. 2 Columns d and e), in the absence of obvious geographic barriers. In this context substrate specialization appears to have fostered diversification ( Fine et al. 2005 , 2014) through parapatric speciation events during the evolution of this New Caledonian *Diospyros* group. Edaphic adaptation has been proposed as one of the main types of parapatric speciation in plants ( Coyne and Orr 2004 ), and has been suggested to have played a key role, for example, in the diversification of Amazonian trees ( Fine et al. 2005 , 2013 , 2014 ).

Grande Terre is a mosaic of soil types ( Maurizot and Vendé-Leclerc 2009 ) and taking the climatic and geological factors together, the island is unusually rich in ecological niches. This likely imposes selection pressures that drive adaptation and isolation, potentially even in the presence of gene flow. In light of our results, the phenotypic characteristics related to substrate adaptation seem evolutionary labile and/or the number of underlying loci for these adaptations is small to allow for relatively frequent niche shifts, but further research is needed to support such conclusions. However, the heterogeneous edaphic conditions have been proposed as the main reason for the high level of endemism found in New Caledonia ( Pillon et al. 2010 ). Isolation by environment has been previously found to be a prevailing evolutionary force across environmental gradients in a wide range of organisms ( Sexton et al. 2014 ), for example in the case of the flora of Lord Howe Island ( Papadopulos et al. 2014 ).

Within the phylogenetic tree of the radiating species of *Diospyros* we have found in different subclades evidence for replicated phenotypic divergence with respect to substrate preference (i.e., ultramafic vs. volcanic soils). Other examples of similar ecological specialists that evolved repeatedly include *Anolis* lizards on the islands of the Greater Antilles ( Losos 2010 ) and African cichlid fishes ( Brawand et al. 2014 ). To further investigate whether natural selection affected a convergent set of loci during parallel evolution ( Purugganan 2014 ), we searched for RAD regions containing SNPs that show high differentiation between two or more sister taxa with the same divergent soil-type preference. Most (92%) of the loci that were successfully annotated indicated that different alleles of the same genes were recruited by natural selection or swept along in the different splits ( Table 2 ). At only 2 of the 26 loci with blast hits did we find that collateral evolution ( *sensu*Stern 2013 ) through sorting of ancestral polymorphism may have played a role in the convergent adaptation, as the same variants were found across different splits. This could also indicate that introgression played only a limited adaptive role across these replicated speciation events. Half of the annotated loci were affected by divergence outside of ORF (open reading frame) regions, potentially indicative of regulatory divergence. A significant portion (35%) of the annotated loci has molecular functions related to ion binding (Supplementary Fig. S6, available on Dryad at http://dx.doi.org/10.5061/dryad.62247 ; Table 2 ); another important category plays a role in transport of elements within or between cells. As the New Caledonian soil types are different in heavy metal content and availability of minerals, these specific loci appear potentially adaptive and therefore involved in edaphic specialization, and not simply linked to regions under selection. It is, however, difficult to argue that this differentiation is responsible for particular speciation events, or if they have evolved subsequently ( Glor 2010 ). A similarly limited number of genomic regions on which positive selection might have acted has also been found in Hawaiian species of *Schiedea* ( Kapralov et al. 2013 ), which exhibit, like the New Caledonian *Diospyros* species, great morphological and ecological variation.

### The Effect of Introgression

It is generally accepted that species divergence can occur in the face of persistent and genome-wide admixture over long periods of time, as shown for example in *Heliconius* butterflies ( Martin et al. 2013 ). Furthermore, it has been proposed that the high speciation rates in radiations may be fuelled by frequent hybridization with transgressive segregation (e.g., Seehausen 2004 ; Gavrilets and Losos 2009 ; Glor 2010 ; Losos 2010 ; Keller et al. 2013 ).

The SplitsTree and Structure analyses provide evidence for considerable admixture among the *Diospyros* species ( Fig. 3 b and Supplementary Fig. S3, available on Dryad at http://dx.doi.org/10.5061/dryad.62247 ). The grouping of different populations of the nonmonophyletic species like *D. minimifolia* and *D. parviflora* has some relationship to provenance. For example, the population of *D. minimifolia* from Gadji (L17, Fig. 1 ) is genetically divergent from the rest of the individuals of this species found in central Grande Terre. This population from Gadji clusters with species from Île des Pins (L25 and L26, Fig. 1 ) and Île Kuebini (L24, Fig. 1 ), which are all in the south of New Caledonia. The SplitsTree network ( Fig. 3 b) clearly indicates regional hybridization as the reason for the split between the *D. minimifolia* populations from the south and those from central portions of the island, which cluster together with northern *D. tridentata* accessions (see also Fig. 2 ). Furthermore, the accessions of *D. parviflora* collected in the central region of New Caledonia are positioned together with the northern accession from L13, closer to other individuals sampled from the northern and central part of the main island. In contrast, the southern populations of *D. parviflora* (i.e., L4 and L19) exhibit evidence of hybridization with southern populations of other species in the SplitsTree network ( Fig. 3 b). This phenomenon of individuals grouping with co-occurring species rather than with populations of the same species from other localities is also found in other organisms (e.g., Martin et al. 2013 ) and may be indicative of genome-wide admixture over long periods of time in many parts of their porous genomes ( Gavrilets and Losos 2009 ; Kane et al. 2009 ). Since most neutral loci are either unlinked or only loosely linked to loci that are under divergent selection, they will be subjected to homogenization by gene flow across closely related sympatric species ( Feder et al. 2012 ). However, given the obvious morphological and ecological differentiation between most of these species we hypothesize as unlikely the swamping of one species with genes of the others ( Rieseberg 2009 ; Butlin et al. 2014 ). Rather, in this context hybridization might have had a positive effect of increasing biodiversity by promoting formation of cryptic species, as evidenced in other groups present in New Caledonia ( Pillon et al. 2009 , 2014 ; Swenson et al. 2015 ).

### Concluding Remarks

Altogether, the species-rich New Caledonian *Diospyros* clade is the result of a rapid adaptive radiation resulting in more than 20 morphologically and ecologically diversified species with low genetic divergence. After surviving a bottleneck associated with the long-distance dispersal event that brought the lineage to the Archipelago, accumulation of genetic variation happened relatively slowly, producing a small effective population size for the new lineage for millions of years. During the first phase of the radiation, this situation resulted in relatively low speciation rates, mainly triggered by allopatry or divergent preference to macrohabitat related to general climatic conditions. After a considerable amount of time (i.e., ca. 6 million years, Fig. 2 ), there was a burst of speciation events, mainly with respect to microhabitat (i.e., edaphic) specialization. Such a delayed burst of speciation is atypical for adaptive radiations, but could be a characteristic of long-generation organisms. Our results suggest that both allopatric diversification (i.e., resulting in regional patterns of diversity) and sympatric/parapatric ecological divergence shaped successive rounds of speciation in the *Diospyros* radiation.

## S upplementary M aterial

Data available from the Dryad Digital Repository: http://dx.doi.org/10.5061/dryad.62247 .

## F unding

This work was supported by the Austrian Science Fund [FWF P22159-B16 to R.S.]. In addition O.P. was partly funded from the Austrian Science Fund [FWF Y661-B16 to O.P.].
